# Insights from Expert Interviews on Navigating the Complexity of Prioritizing Chemicals for Human Biomonitoring in Latvia

**DOI:** 10.3390/toxics13090715

**Published:** 2025-08-25

**Authors:** Linda Matisāne, Lāsma Akūlova, Ilona Pavlovska, Monta Matisāne, Ivars Vanadziņš

**Affiliations:** 1 Institute of Occupational Safety and Environmental Health, Rīga Stradiņš University, Dzirciema 16, LV-1007 Riga, Latvia; monta.matisane@rsu.lv (M.M.); ivars.vanadzins@rsu.lv (I.V.); 2 Laboratory of Hygiene and Occupational Diseases, Institute of Occupational Safety and Environmental Health, Rīga Stradiņš University, Dzirciema 16, LV-1007 Riga, Latvia; lasma.akulova@rsu.lv (L.A.); ilona.pavlovska@rsu.lv (I.P.)

**Keywords:** human biomonitoring, national program, chemical prioritization, Hanlon methodology, environmental health, public health monitoring

## Abstract

Human biomonitoring (HBM) is a vital tool for assessing chemical exposure in populations and informing evidence-based public health policy. For smaller countries such as Latvia, establishing a national HBM program presents specific challenges, including limited prior experience, national data gaps, and resource constraints. This study explores the expert experiences and reflections gathered during the development of Latvia’s national HBM chemical prioritization process. Semi-structured interviews were conducted with eight experts who were directly involved in evaluating and selecting substances for inclusion in the program. The focus of this study is not on the outcomes of the prioritization itself—published elsewhere—but rather on the strategies applied, challenges encountered, and lessons learned in navigating the prioritization process. A qualitative content analysis identified several key themes, including limitations in data availability, institutional coordination challenges, differences in expert opinion, and the complexity of adapting international methodologies to the national context. Despite these obstacles, the process benefitted from interdisciplinary collaboration, iterative methodological refinement, and the strategic use of international frameworks. The findings offer practical insights for countries with limited resources that are initiating or refining their national HBM programs. This study highlights the importance of national data infrastructure, stakeholder engagement, and tailored methodological approaches to ensure an effective and context-sensitive prioritization process.

## 1. Introduction

According to the World Health Organization (WHO), human biomonitoring (HBM) is a reliable tool for assessing human exposure to chemicals from different sources, by different pathways, and during certain periods of life through direct measurement of concentrations of chemical pollutants or their metabolites in human fluids and tissues [1]. Data obtained through HBM can inform current and future understanding of exposure and health impacts and serve as a useful tool to further enhance the evidence base, allowing timely and targeted policy interventions and risk management [2].

It has been recognized that HBM is usually based on national HBM programs that are complex processes with several stages/phases that require multidisciplinary expertise, involving scientists, risk assessors and managers, epidemiologists, toxicologists, laboratory specialists, lawyers, and journalists, among others [3]. While some national HBM programs have a long history and some are relatively new, many countries are still in the process of establishing their own systems [3,4].

Latvia recently completed the initial step in establishing its national HBM program by prioritizing chemicals and the technical process, as well as the results, are described in detail in our previous publication. This process consisted of two major steps—identification of chemical substances/substance groups and prioritization. The identification of prioritized chemical substances and substance groups was conducted through a series of substeps. Initially, the Ministry of Health, within the framework of the national research program, predefined three broad priority categories: pesticides, heavy metals, and persistent organic pollutants [5], reflecting the state’s recognized research needs. Due to the absence of a comprehensive national exposure database, Latvia initially relied on existing international prioritization frameworks-the particular chemicals were further researched with a thorough review of existing priority chemical lists developed under other international HBM initiatives, particularly HBM4EU [6]; and the German HBM program [7]. These served as a foundational reference to ensure alignment with broader EU priorities. While these frameworks provided a valuable reference point, there was a recognized risk of underrepresenting local exposure conditions and national specific risks if not supplemented with contextual information. Thus, the prioritization was also driven by national expert input, taking into account the environmental health context specific to Latvia, data availability, regulatory needs, and feasibility of implementation. Experts adapted and expanded the criteria to reflect local relevance—such as the prominence of certain pesticides in Latvian agriculture, the national discourse on chemical safety, and practical considerations for HBM under existing infrastructure. This ensured that the resulting list was not a replication of EU priorities, but a context-specific selection tailored to public health needs in Latvia [8].

During this process (described in a previous publication), 318 chemical substances were reviewed, 130 of which were shortlisted and assessed using an adaptation from the original Hanlon equation [9]. In the adapted method, the national expert panel with a background in chemistry, public health, medicine and environment:

— retained component A (problem size-percentage of exposed population; use of 10% in weighting);
— expressed component B (severity of the problem) with two components:
  ○ component B (hazardous properties-carcinogenicity, mutagenicity, reproductive toxicity, developmental toxicity, endocrine activity, STOT RE (systemic toxicity after repeated exposure), neurotoxicity, immunotoxicity, respiratory sensitization, skin sensitization; use of 30% in weighting);
  ○ component C (exposure characteristics-persistency and/or bioaccumulation potential, sales in the EU or, where possible, sales in Latvia (tons per year), exposure routes, passage of placental barrier, exposed population, level of concern of the exposure; use of 30% in weighting).

For further adaptation to the national context, component D (national significance; use of 15% in weighting) and component E (public interest; use of 15% in weighting) were added to the equation. Based on the literature review, the method, including weights and the weighting process, was suggested by experts and approved by a consensus from the members of the National Human Biomonitoring Council. More detailed adaptation process, amendment forces for the developed methodology, involvement of the national Human Biomonitoring Council and relevant national state authorities as well as specific criteria for each component, identification and shortlisting of chemicals and the final methodology for scoring has been published earlier [8]. Key aspects of prioritization are provided in Table 1.

This evaluation resulted in the identification of 30 high-priority substances across various categories, forming the foundation for the HBM4LV program. The experience highlighted that chemical prioritization for national HBM programs is a crucial but underexplored area, especially for smaller nations with limited experience and resources. While several countries have established frameworks for prioritizing chemicals, there remains a significant gap in published methodologies tailored to address the unique challenges faced by nations with limited experience and resources like Latvia. This is in line with the WHO-in its meeting “Health-related priorities in chemical safety–focus on human biomonitoring and poison centres”, held in Bonn, Germany, 12–13 October 2022, has highlighted the need for an outline of specific needs and considerations for countries with limited resources [10].

It has been reported earlier that as with any new project and activity, the establishment and implementation of national HBM programs have to overcome challenges and barriers like the lack of political will, low public awareness, lack of availability of information, data and databases, limited resources and variations in needs and situations [10,11]. In our case, the lack of prior experience and published guidance for prioritization of chemicals for national programs posed considerable challenges for experts involved in this process, requiring them to adapt and innovate without a tested roadmap. The absence of standardized approaches highlights the broader importance of developing and documenting methodologies that account for national priorities, data limitations, and regional differences in chemical exposure. Thus, we decided to publish both—(1) the results of the entire prioritization with the approved list of chemical substances, including pesticides, metals, persistent organic pollutants and others [8] and (2) the challenges we encountered and the lessons we learned. The focus of this study is not on the outcomes of the prioritization itself—published earlier [8]—but rather on the strategies applied, challenges encountered, and lessons learned by national experts in navigating the prioritization process. By documenting these insights, we aim to contribute to the broader discussion on developing effective, context-specific approaches that can guide other nations in similar endeavors.

## 2. Materials and Methods

Semi-structured interviews with experts involved in the identification and prioritization of chemical substances and groups of chemical substances for the establishment of the HBM process in Latvia were conducted to gather their feedback on the process, challenges they met during the process and the needed improvements that could be implemented to facilitate similar processes if they are carried out in other countries.

### 2.1. Study Design

For the evaluation process of chemicals, three different teams of researchers representing academia were established to assess chemical substances/chemical substance groups. Each group consisted of at least two researchers with different backgrounds–one of them had a background in chemistry, another one-in public health or medicine. The evaluation of chemicals in this study was conducted exclusively by the national academic experts, who brought their experience in scientific assessment, evidence synthesis, and preparation of research-based documentation. Their methodological knowledge and independence ensured an objective and evidence-informed prioritization process. Although relevant state authorities were not directly involved in the evaluation step, they had distinct responsibilities in reviewing the results, providing feedback, and contributing to national-level interpretation of findings. In addition, concerns raised by non-governmental organizations (NGOs) were taken into account during the prioritization process, and opportunities for NGOs to comment and provide input on the selection of substances were made available, ensuring that civil society perspectives were reflected in the final outcome. These involvements, as well as the use of international experience, are described in detail in our previous article.

The involved experts who accepted the invitation to participate in the interviews have the following backgrounds:

— Expert 1—public health, occupational health and safety;
— Expert 2—chemical engineering;
— Expert 3—chemistry, occupational and environmental medicine, toxicology;
— Expert 4—chemistry;
— Expert 5—public health, occupational and environmental medicine;
— Expert 6—chemistry;
— Expert 7—occupational health and safety;
— Expert 8—occupational medicine.

The invitation process to participate in the interviews was based on voluntary personal invitations sent to the emails of all experts who were involved in the valuation process of chemicals and drafting of the article describing the process and the results, with the option to provide answers during an online meeting or in writing. Ten experts were invited, out of which eight agreed to the online option, and two declined to share their opinion.

Before the interviews, participants were fully informed about the purpose of the interviews, and, therefore, electronic consent was obtained.

### 2.2. Guidelines Used for the Interviews

All interviews followed a standardized procedure. Structured research guidelines with logically proceeding questions were developed at the initial phase of the study. The translated guidelines are included in Appendix A, Table A1. The themes and questions were drafted by the management of the research team, led by an experienced researcher (I.V.). According to the structured guidelines, the following themes were covered: (1) prioritization process of chemical substances and groups of chemical substances; (2) sources of information and digital tools; (3) main challenges.

### 2.3. Process of Interviewing

Online interviews were organized using an online platform (Zoom), and the questions were asked by an experienced and trained researcher (I.V.) who was not involved in the original process of evaluation of chemicals. A PowerPoint presentation was used to share the slides with questions when the corresponding themes were covered to facilitate online conversation. The conversations were recorded after obtaining the experts’ permission; the recordings were used to facilitate transcription and ensure that the information was accurately matched. Recordings are stored securely in accordance with the data protection rules of the Rīga Stradiņš University. The length of the transcribed recordings was 185 min, averaging 23 min per expert. In addition, some experts wrote comments in the chat function, which were incorporated as quotes in the transcribed text.

### 2.4. Data Analysis

Anonymized transcripts were created by the transcriber who was not involved in the evaluation process (M.M.). He manually redacted the expert’s identity and replaced their names with labels such as Expert 1, Expert 2, Expert 3, and so on. During the interviews, one of the experts who did not agree to participate in the interviews was also mentioned during the discussions. His name was also replaced with labels (Expert 9, with a background in environmental toxicology and environmental sciences). Expert 10 (with a background in public health and occupational health and safety) was not mentioned in the discussions; therefore, no labels were used for him.

The transcriber was not involved in prioritizing chemical substances or groups of chemical substances, nor in the subsequent analysis of the findings. Rigorous and systematic conventional content analysis, encompassing both coding and interpretative tasks, was independently performed by two coders (L.M. and L.A.). One of the coders was not directly involved in evaluating the chemicals, while another was thoroughly involved in both amending the Hanlon methodology and assessing the substances. This combination ensured both objectivity and contextual depth in the analysis, as one coder brought an independent perspective while the other contributed in-depth knowledge of the evaluation process and methodological framework.

Initially, both researchers collaboratively reviewed the anonymized transcripts and developed preliminary codes grounded directly in the data rather than theoretical frameworks. This approach enabled an unbiased evaluation that aligned with the research question. Subsequently, the researchers independently coded the transcripts and later compared their analyses, reaching a consensus on the final set of codes (for details, see Appendix A, Table A2):

— Main sources of information (ECHA, scientific literature, national HBM programs of other countries, data from the HBM4EU project, International Agency for Research on Cancer, national data);
— Main challenges (time constraints, low availability of national data, insufficiently clear opinion of state authorities, complex and huge work, quality control, changes in the methodology, insufficient specific knowledge, objectivity);
— Positive aspects (team approach, availability of the written methodology and supporting templates, obtained new skills and knowledge, Use of artificial intelligence);
— Suggestions for improvements (kick-off training and regular updates, previous involvement in international projects, change management, clear state framework).
— During the coding process, both researchers identified and recorded relevant text segments (quotes), which were later discussed and incorporated as anonymized examples to illustrate the diversity of responses. Only the most representative quotes were included in the section “Results” (in English). In contrast, additional supporting quotes were organized into a table in Appendix A, Table A2 (in Latvian and English).

### 2.5. Use of AI-Assisted Tools in Manuscript Preparation

Portions of this manuscript, including the state of importance, parts of the discussion, and the abstract, were prepared with the assistance of ChatGPT-4 (OpenAI, San Francisco, CA, USA). However, this assistance does not meet the criteria for intellectual contributions required for authorship. The authors have thoroughly reviewed and revised the content to ensure its accuracy and alignment with academic standards. Full responsibility for the final content of this manuscript lies with the authors.

## 3. Results

The process of prioritizing chemical substances and groups of chemical substances for Latvia’s national HBM program provided significant insights into the challenges and opportunities faced by countries with limited resources. Our results show that key obstacles included limited national data, insufficient guidance from state authorities, and diverse expert opinions. A summary of the prioritization outcomes, including baseline, process characteristics, and risks, is provided in Table 1.

### 3.1. Challenges Encountered

#### 3.1.1. Limited National Data Availability

A major challenge in the prioritization process was the scarcity of national data, which limited the ability to make robust, evidence-based decisions. Unlike larger nations or those with established HBM programs, Latvia’s lack of comprehensive datasets required reliance on fragmented and incomplete information. As Expert 6 remarked, “We cannot fully equate [the situation in our country] with other European countries that have different geographical conditions or certain processes taking place.” This gap created additional challenges in contextualizing international data for the local environment. Similarly, the sample sizes in available studies were often insufficient to draw strong conclusions, as noted by Expert 3: “There is limited data, the sample size [used in implemented studies] is small...” The absence of consistent longitudinal data further complicated the process, underscoring the critical need to establish a robust national data infrastructure.

#### 3.1.2. Insufficiently Clear Guidance from State Authorities

Another significant barrier mentioned by the experts during the interviews was the lack of clear and actionable guidance from state authorities. Experts frequently found themselves navigating ambiguous recommendations, which added complexity to an already challenging process. Expert 6 highlighted this issue: “The information was incomplete and insufficient; more like a source of general information.” This ambiguity led to inefficiencies, as the team had to spend considerable time interpreting and supplementing incomplete information. The vagueness of state recommendations was particularly problematic when addressing groups of broad chemical categories. Expert 5 highlighted the lack of specificity: “Those letters [received from the state authorities]... did not provide anything useful to me, because... saying that phthalates [in general] need to be identified when there are about 120 or so phthalates, [without specifying which ones have high risk].” This experience underscores the importance of providing precise, well-defined guidelines to facilitate efficient prioritization and resource allocation.

#### 3.1.3. Diverse Expert Opinions

The prioritization process was further complicated by the diverse interpretations and opinions among experts, particularly when evaluating chemicals with limited or conflicting data. Expert 6 articulated the challenge: “The hardest part… was probably when the information was not straightforward or when there were contradictory studies.” Diverging perspectives on the weight of different criteria often led to lengthy deliberations, making it difficult to achieve consensus. For example, while some experts advocated for prioritizing substances based on international frameworks like HBM4EU, others emphasized the need to address region-specific exposure patterns. This divergence required iterative adjustments to methodologies and scoring systems to reconcile differing viewpoints.

#### 3.1.4. Complexity and Volume of Work

The scale and intricacy of the prioritization tasks presented additional challenges. The adapted Hanlon methodology required detailed evaluations of multiple criteria, including hazard properties, exposure characteristics, and national significance. Expert 1 reflected on this complexity, stating, “Initially, we wanted to keep it [the methodology or chemical prioritization] simple, but... we realized that the simple approach was not enough.” As the team delved deeper into the process, the volume of work expanded significantly, necessitating extensive collaboration and iterative refinements.

### 3.2. Strategies and Positive Outcomes

#### 3.2.1. Collaborative Approach

A key strength of the prioritization process was the effective collaboration among team members, which helped distribute the workload and optimize the use of available expertise. Expert 8 described the collaborative dynamics: “We worked as a team, where one person focused more on development, another on writing, and someone else handled other tasks”. This division of labor ensured that the team could address the diverse aspects of the prioritization process, from data collection to methodological application and documentation. By fostering open communication and shared responsibility, the team was able to navigate challenges effectively, even in the face of limited resources. The collaborative approach not only streamlined the workflow but also enhanced the overall quality of the outputs through cross-checking and peer reviews.

#### 3.2.2. Utilization of International Frameworks

In the case of Latvia, reliance on international frameworks such as HBM4EU was instrumental in establishing a solid foundation for the prioritization process. Leveraging the comprehensive datasets and methodologies developed through HBM4EU allowed the team to align its efforts with broader European objectives while tailoring the information to Latvia’s specific context. As one expert noted, “I took all the substances that were already included in the previous HBM4EU project and used them as a basis for our evaluations”. This strategic alignment facilitated access to robust data and proven methodologies, significantly enhancing the credibility and efficiency of the prioritization process. In addition, Expert 3 noted the value of these networks: “My approach was quite simple. I took all the substances that were already included in the previous HBM4EU project, the metals that had already been prioritized, or the metals that had been discussed as ones that should be examined. And that was essentially the starting point [for prioritization in our national context]…” This integration not only improves efficiency but also ensures alignment with broader European goals.

#### 3.2.3. Iterative Methodological Refinements

The continuous refinement of the adapted Hanlon methodology was another positive aspect of the process. As Expert 7 remarked, “I was glad that the methodology was… updated, refined, and adjusted to better suit our needs.” This iterative approach allowed the team to address emerging challenges, incorporate feedback from stakeholders, and enhance the accuracy and applicability of the prioritization criteria. The flexibility to adapt the methodology in real-time was particularly valuable in navigating the complexities of chemical assessment.

### 3.3. Lessons Learned

#### 3.3.1. Need for Comprehensive National Data

The lack of robust national data emerged as a significant barrier during the prioritization process. Developing a comprehensive data infrastructure, including data from national sources and research, is critical to overcoming this challenge and ensuring the long-term success of the HBM program. As Expert 1 highlighted, “I had the great advantage of having a study to consider. We had the results on pesticides from the HBM4EU SPECIMEN study, and we decided to evaluate those pesticides that were detected in at least 15% of the population.” This expert also emphasized, “Another advantage was that official statistics on sales volumes are available, which helped us evaluate certain chemicals,” highlighting the potential value of well-maintained and accessible datasets.

#### 3.3.2. Importance of Clear Guidance

The need for specific and actionable guidance from state authorities was another key lesson from the process. Expert 6 emphasized the importance of clarity: “The information was incomplete and insufficient...” Similarly, Expert 5 pointed out the inefficiency caused by vague recommendations, stating, “Those letters [from the state authorities] ... did not provide anything useful to me”.

#### 3.3.3. Value of Specialized Expertise

Involving additional experts with specialized knowledge in fields such as toxicology and chemistry was identified as a crucial factor in enhancing the quality of the prioritization. Expert 1 reflected on this, stating, “In chemistry and toxicology, we had an invited expert—Expert 9—who made some comments, but ... yes, if there had been someone else with solid expertise to review it further, it would have been even better” (Expert 9 did not agree to participate in interviews).

## 4. Discussion

One of the key challenges during the prioritization process was the limited availability of national data, which restricted the ability to base decisions on solid evidence. National exposure data were available only for pesticides and even then, only for five of the shortlisted 16 pesticides (less than 4% of all substances in the shortlist) [8]. Furthermore, these data were based on a urinary suspect screening approach [12]. Due to the absence of comprehensive datasets, the team had to depend on partial or fragmented information. This made it difficult to adapt international data to Latvia’s specific context. Additionally, many existing studies were based on small sample sizes, reducing the reliability of conclusions. The lack of consistent, long-term data further emphasized the pressing need to build a robust national data system to support future HBM initiatives. When compared to research efforts in countries like Germany or France [6,7], where long-term HBM programs provide comprehensive datasets for decision-making, Latvia’s situation highlights the challenges faced by smaller nations with limited resources and experience. Such experience has been highlighted by researchers in other smaller countries, e.g., Ireland [4].

Despite the WHO’s emphasis on the critical role of state authorities and policymakers in supporting HBM programs [3], experts in Latvia identified the lack of clear, actionable guidance from national institutions as a major barrier. Ambiguous and overly general recommendations complicated the process, requiring the team to spend additional time interpreting and supplementing missing information. This was particularly problematic when broad chemical categories were referenced without specific prioritization criteria. The experience highlighted the importance of well-defined, targeted guidance to ensure an efficient and coherent prioritization process.

The prioritization process was also challenged by differing expert opinions, especially when data were limited or inconsistent. Variations in how the criteria were interpreted led to extended discussions and made consensus difficult. While some experts favored alignment with international frameworks such as HBM4EU, others prioritized chemicals based on local exposure concerns. These differing viewpoints necessitated ongoing methodology and scoring system adjustments to reach balanced decisions. Compared to well-resourced nations, where comprehensive datasets and clear evaluation criteria often reduce such conflicts, Latvia’s reliance on expert judgment highlighted the need for very strong methodological frameworks to guide decision-making.

The complexity and scope of the prioritization process, driven by the detailed criteria of the adapted Hanlon methodology, quickly exceeded initial expectations and required substantial teamwork and continuous refinement. When compared to studies conducted in countries with extensive resources and established frameworks, such as the U.S. National Health and Nutrition Examination Survey [13], the German Environmental Survey [7], the Latvian team faced unique challenges in managing workload with limited expertise and funding. These comparisons highlight the need for smaller nations to adopt streamlined processes while maintaining methodological rigor to ensure the success of their HBM initiatives.

Effective teamwork and clear division of responsibilities enabled the prioritization team to manage complex tasks efficiently, optimize limited resources, and ensure the overall quality of the process. This emphasis on teamwork aligns with best practices observed in larger HBM initiatives, demonstrating that even resource-constrained nations can benefit from structured, cooperative strategies.

Participating in multinational, primarily global, initiatives provide significant political incentives and opportunities for capacity-building and cost- and data-sharing. It also enhances the overall impact and ensures greater sustainability of the program [3,4]. The integration of international frameworks underscores the importance of collaboration across borders, especially for smaller nations with limited resources. Such partnerships not only bolster technical and methodological capacity but also enable the adoption of best practices in chemical monitoring, ultimately contributing to more effective and sustainable national programs. For example, setting up a collaboration with international laboratories that can support the analysis of the most relevant chemicals and their metabolites and/or biomarkers, in cases where the national laboratories have limited capacity and expertise for the required tests [2].

The iterative refinement of the methodology allowed the team to adapt to challenges, integrate stakeholder feedback, and improve the relevance and precision of the prioritization process. Compared to static methodologies employed in some other HBM programs, the iterative process used in Latvia demonstrates how adaptability can drive better outcomes, particularly when resources and data are limited.

The absence of robust national data was a major obstacle, underscoring the need to establish a comprehensive data infrastructure to support evidence-based prioritization and ensure the sustainability of the HBM program. Expanding and standardizing data collection efforts would enable more accurate and reliable assessments in the future. Countries with established HBM programs, such as Germany and Canada, have demonstrated the value of detailed, longitudinal datasets [6,7,14,15]. For countries willing to establish their national HBM programs, investing in similar infrastructure would not only improve the prioritization process and be in line with the recommendations of the WHO [3] but also support evidence-based policy decisions and public health interventions.

Another key lesson was the importance of receiving clear and specific guidance from state authorities, as vague recommendations hindered the efficiency and focus of the prioritization process. Other countries have also emphasized the high priority of guidance from relevant state authorities [16]. Clear, well-defined guidance would streamline the prioritization process, reduce ambiguities, and ensure better alignment with national priorities. This lesson underscores the need for stronger communication channels and structured frameworks between state institutions and research teams, enabling more effective collaboration and resource utilization.

Specialized expertise in fields such as toxicology and chemistry was seen as critical to enhancing the scientific accuracy and overall quality of the prioritization process. The inclusion of domain-specific expertise would not only improve the technical rigor of the assessments but also facilitate more nuanced interpretations of complex chemical data. Investing in capacity-building initiatives, such as training programs and partnerships with international experts, could address this gap and strengthen the overall effectiveness of the HBM program. This lesson is particularly relevant for smaller nations, where resource constraints often limit access to a diverse pool of expertise.

This study has several limitations that should be acknowledged. First, the qualitative findings are based on interviews with a relatively small number of experts in one country (*n* = 8), which may not fully capture the diversity of perspectives involved in national HBM program development. While participants had diverse backgrounds and key stakeholders were involved in different stages of the assessment process, the weak interest and perspective from policymakers or representatives of state authorities may have limited the comprehensiveness of institutional viewpoints. Second, the prioritization process was influenced by the availability and quality of existing data, which were limited in scope and coverage. The absence of robust national datasets constrained evidence-based decision-making and may have introduced bias toward substances already studied or prioritized in international frameworks. Third, although the adapted Hanlon methodology provided a structured approach, the absence of a validated national scoring system and the need for iterative adjustments may have affected consistency and reproducibility. The methodological refinements made during the process, while practical, could limit the comparability of results across contexts. Lastly, since this study focuses on Latvia’s specific national context, some findings may have limited generalizability. However, the documented challenges and lessons learned can still offer valuable guidance to other countries facing similar constraints in establishing HBM programs.

Despite these limitations, we believe that this article provides valuable insights for other countries that are beginning the establishment of their HBM programs.

## 5. Recommendations

### 5.1. Development of National Data Infrastructure

Establishing a centralized and accessible national data infrastructure is crucial and urgent for addressing the gaps in chemical exposure data that hinder the prioritization process. A robust data infrastructure would provide the foundation for evidence-based evaluations and policy decisions. Investing in comprehensive data collection mechanisms and integrating these with international systems like HBM4EU would enhance data comparability and improve the overall effectiveness of the national program. By building this infrastructure, the country developing its national HBM program can align with best practices observed in countries like Germany and Canada, where longitudinal datasets significantly contribute to public health monitoring and policy-making [6,7,14,15]. Moving forward, establishing a local exposure database, enhancing chemical monitoring networks, and encouraging integration of biomonitoring efforts with environmental and product-use registries are crucial steps. Such initiatives would reduce reliance on international assumptions and allow more precise modeling of exposure scenarios and health risks in Latvia.

In addition, international methodological guidance should be developed by collaborative scientific bodies and agencies with expertise in HBM and chemical risk assessment to transparently manage uncertainties and assumptions when direct national data are lacking. This might exclude neglecting the specific exposure scenarios and risks particular to the local area. Thus, the experience highlighted the urgency of investing in national data generation to support robust and context-specific prioritization. In cases of insufficient data, structured expert elicitation and transparent uncertainty handling were essential.

### 5.2. Implementation Roadmap and Policy Recommendations

To ensure the successful implementation of a national HBM program, a clear roadmap is essential. This should include dedicated and sustainable funding mechanisms, ideally through multi-annual state budget allocations and integration with EU co-funded initiatives such as HBM4EU and PARC. A phased timeline is also recommended—starting with pilot biomonitoring of a few high-priority substances (e.g., glyphosate, lead) within 1–2 years, followed by expansion to a broader panel over a five-year horizon, and then reviewing the list on a regular basis (e.g., every two years) [17,18]. The roadmap should also define institutional responsibilities, stakeholder engagement strategies, and alignment with ongoing policy frameworks on chemical risk assessment and public health monitoring. It is essential to highlight that for the further implementation of the national HBM programs some other aspects should be taken into account, like available analytical methods, the cost of specific tests or test suites, additional costs linked to transportation of samples [16], the cooperation and engagement of the individuals being sampled, etc. [19].

### 5.3. Enhance Collaboration Across Sectors

Collaboration between researchers, policymakers, and international networks like HBM4EU was a cornerstone of the prioritization process and should be further strengthened. Expanding this collaborative approach to include state institutions, non-governmental organizations, and industry representatives would ensure a holistic perspective and a more inclusive prioritization process. Engaging with international initiatives such as HBM4EU provides access to shared expertise, resources, and comparative data, which are invaluable for smaller nations.

### 5.4. Improve Methodological Guidelines

The need for clear and standardized methodological guidelines emerged as a critical lesson from the prioritization process. While iterative adjustments to the adapted Hanlon methodology allowed for flexibility, it also caused inefficiencies in the prioritization process. Future efforts should focus on developing robust, detailed protocols from the outset. These guidelines should include well-defined scoring criteria, transparent prioritization processes, and standardized data collection methods tailored to the country’s context. By adopting such frameworks, the HBM program can achieve greater consistency and reproducibility, reducing the reliance on ad hoc adjustments during the prioritization process.

### 5.5. Allocate Sufficient Resources

Sufficient resources—both financial and human—are essential to manage the complexities of chemical prioritization effectively. This underscores the need for realistic timelines and adequate funding to support comprehensive analyses and the development of expertise. Investing in training programs, expert recruitment, and data collection infrastructure would enhance the overall quality and efficiency of the HBM program. Providing adequate evaluation time would also enable the team to address data gaps more thoroughly and incorporate emerging findings, ensuring that the prioritization process remains scientifically rigorous. In addition, sufficient resources can provide the possibility for experienced international experts to support the prioritization process in countries lacking national expertise [20].

## 6. Conclusions

Our experience demonstrates how strategic collaboration, methodological flexibility, and the integration of international best practices can overcome diverse challenges of the prioritization process of chemicals within the HBM process, including resource limitations. The process exemplifies how smaller nations can establish effective HBM programs by utilizing multidisciplinary teamwork, iterative methodological refinements, and alignment with external frameworks.

This case study provides valuable insights into the process of chemical prioritization for other resource-constrained countries aiming to implement HBM initiatives. It underscores the significance of centralized data infrastructure, clear methodological frameworks, and cross-sector collaboration in achieving sustainable outcomes. Latvia’s journey could serve as a replicable model for building national capacities in chemical exposure monitoring in humans, contributing to global efforts to enhance public health protection.

## Figures and Tables

**Table 1:** Key aspects of prioritization of chemical substances and groups of chemical substances for the HBM program in Latvia.

Component	Area
Outcome Statements	- Identified 30 high-priority chemical substances using an adapted Hanlon methodology - Prepared a solid foundation for Latvia’s National Human Biomonitoring Program (HBM4LV)
Baseline	- Initial review of 318 chemical substances, with 130 shortlisted for detailed evaluation - Limited national data and resources posed challenges
Process	- Prioritization performed by a team of 10 experts - Adapted Hanlon methodology utilized for evaluation of chemicals and groups of chemicals - Collaboration with international frameworks like HBM4EU ensured methodology alignment - Approval from the National Human Biomonitoring Council
Assumptions/Risks	- Limited availability of comprehensive national datasets - Diverse expert opinions and insufficient guidance from state authorities - Dependence on fragmented and incomplete information

## Data Availability

No new data were created or analyzed in this study. Data sharing does not apply to this article.

## Appendix A

**Table A1:** Semi-structured expert interview guidelines (the scheduled length of interviews–approx. 30 min).

Introduction
Acquaintance with the interviewer, the purpose and tasks of the interview. - Greetings—the interviewer introduces himself/herself (given name, surname, RSU Institute for Occupational Safety and Environmental Health, if necessary). - The purpose of the interviews is to find out the opinion of the experts involved in the assessment of chemical substances and groups of chemicals on the assessment process. Basic information: - Thank you for being able to join us today; - There are no “right” or “wrong” answers; - Recording of the interview to provide opportunities to summarize the results of the interviews; - Ensuring confidentiality (data will only be used in an aggregated form); - Please turn off your cell phones (at least put on silence).
Evaluation process
— How do you evaluate the process of developing the methodology? Do you think there were better/other ways to evaluate and prioritize chemicals? — Where did you start the evaluation? How did you identify the specific substances to be included in the evaluation? — At what point did you take into account letters from government agencies? — If the work had to start again from scratch, what would be done differently? — In your opinion, can information be collected by one and Hanlon calculated by another? Do you have any suggestions for further development -how best to share works?
Used sources of information and digital tools
— What were the main sources of information? — Did you use artificial intelligence (ChatGPT)? — How did you use? Paid/free version? What did you ask? — With today’s experience, how to use differently? — Why didn’t you use?
Challenges
— What was the hardest part of the evaluation process? Did you have “jams”? — Would you agree to do it again? What would you do differently this time? — What were the shortcomings of the process? — Was there any specialist missing from the team? — What did you like about the process? — One tip if any country started from scratch creating a biomonitoring program?
Final remarks
Would you like to add something else in relation to the evaluation process? - Thanks for participating! - Privacy reminder!

**Table A2:** Code system and quotes.

Codes	Number	Quotes (Latvian)	Quotes (English)
Main sources of information			
ECHA (European Chemical Agency)	6	Eksperts 2: Es laikam vairāk skatījos ECHA mājaslapu un no turienes ņēmu, bet dažreiz biju skatījusies tajā Amerikas, es neatceros, kādā datubāzē, ...	Expert 2: I think I mostly looked at the ECHA website and took information from there, but sometimes I checked that American database as well, although I do not remember which one.
Scientific literature	5	Eksperts 7: Protams, publikācijas datu bāzēs, piemēram, PubMed ar atslēgas vārdiem meklējot.	Expert 7: Of course, in publication databases, such as PubMed, by searching with keywords.
		Eksperts 5: Bet arī ja ECHA nav tā bīstamība, tas nozīmē, ka viss ir kārtībā. Bet es tam nepiekrītu. Ja ir pētījumi, ja ir vairāki pētījumi, tad tas arī varētu tikt iekļauts kaut kādā izvērtēšanā, bet es piekrītu, ka jāobjektivizē. Ja tas ir kāds pamatojums jāmeklē, tad tas ir oficiāls pamatojums. … Jo pētījumi iet uz priekšu, viņi straujāk parāda kaut kādas problēmas.	Expert 5: But even if ECHA does not list the hazard, it does not mean everything is fine. I do not agree with that. If there are studies, if there are multiple studies, then those could also be included in some sort of evaluation, but I agree that it needs to be made objective. If some justification is needed, then it must be an official justification. … Because research progresses, and it often highlights certain issues more quickly.
		Eksperts 6: Grūtākais.... laikam bija …, kad nebija tā informācija viennozīmīga. … Tad, kad Tu sāc novērtēt pēc zinātniskās literatūras. Uz svaru kausiem likt–augsta, zema vai vidēja, saprast, vai tas pētījums ir iespējams korekts vai nav korekts. Salīdzināt ar kādu citu, kas ir pētījis līdzīgu, bet viņam ir citādi rezultāti. Tad kuram ticēt un kuru likt?	Expert 6: The hardest part… was probably when the information was not straightforward. … When you start evaluating based on scientific literature. Weighing—high, low, or medium—and trying to understand whether a study is possibly accurate or not. Comparing it with another study that has researched something similar but has different results. Then, whom to trust and which one to choose?
National human biomonitoring programs of other countries	3	Eksperts 5: Un otra lieta bija Kanādas biomonitoringa programma, kas viņiem gadiem iet un Amerikas biomonitoringa programma. Protams, varētu teikt, ka tas ir cits kontinents, citi, nezinu, produkti, paradumi, ieradumi, likumdošana, bet diemžēl tas bija vismasīvākais, vispārbaudītākais dinamikā un nezinu izpētītākais, uz ko paļauties, kuras vielas ir apritē, kuras nē, kuras ir aktuālas, kurš ne.	Expert 5: And the other thing was the Canadian biomonitoring program, which has been running for years, and the American biomonitoring program. Of course, one could say it is a different continent, with different products, habits, customs, and legislation, but unfortunately, it was the most extensive, dynamically tested, and, I would say, the most thoroughly researched source to rely on for understanding which substances are in circulation, which are not, and which are relevant or not.
Data from the HBM4EU project	3	Eksperts 3: Mana pieeja bija pavisam vienkārša. Paņemt visas tās vielas, kas jau bija iepriekšējā projektā HBM4EU, tos metālus, kas jau bija prioritizēti, vai tos metālus, par ko bijusi runa, ka tos vajadzētu skatīties. Nu un tas principā bija izejošais informācijas avots, tīri tā pragmatiski, lai pēc tam būtu ar ko salīdzināt, ja mēs skatāmies, lai varam salīdzināt ar citiem, tad lai tās ir konkrētās Eiropas kontekstā nosauktās aktuālās vielas.	Expert 3: My approach was quite simple. I took all the substances that were already included in the previous HBM4EU project, the metals that had already been prioritized, or the metals that had been discussed as ones that should be examined. And that was essentially the starting point, purely pragmatic, so that later there would be a basis for comparison. If we are looking to compare with others, then it is important to focus on the specific substances identified as relevant in the European context.
		Eksperts 5: Es ņēmu piemēru no tā, kas jau ir darīts Eiropas lielajā biomonitoringa projektā.	Expert 5: I took an example from what has already been done in the large European biomonitoring project.
International Agency for Research on Cancer	2	Eksperts 7: Es diezgan daudz izmantoju arī Starptautiskās vēžu izpētes aģentūras datu bāzes un materiālus, ļoti daudz tur skatījos.	Expert 7: I also used the International Agency for Research on Cancer databases and materials quite extensively; I looked there a lot.
National data	2	Eksperts 1: Man bija tā foršā lieta, ka mums bija pētījums, ko ņemt vērā. Ka mums bija tas pesticīdu pētījums HBM4EU SPECIMEN study, un mēs pieņēmām lēmumu, vērtēt tos pesticīdus, kas bija konstatēts vismaz 15% populācijas.	Expert 1: I had the great advantage of having a study to consider. We had the results on pesticides from the HBM4EU SPECIMEN study, and we decided to evaluate those pesticides that were detected in at least 15% of the population.
		Eksperts 1: Otrs labums, kas bija, bija tas, ka ir pieejama oficiāla statistika par pārdošanas apjomiem, ir Excelis, nolādē, apskaties un izrēķini vidējo, apskaties tendenci. Ļoti superīgi, ir tāda statistika pieejama par šo vielu grupu.	Expert 1: Another advantage was that official statistics on sales volumes are available. You can download an Excel file, review it, calculate the average, and analyze the trend. It is great that such statistics are available for this group of substances
		Eksperts 5: Un tie informācijas avoti man arī bija tāpat kā kaut kas jau minēja tie [Valsts] Vides dienesta minētie dati ūdenī, augsnē, pārtikā.	Expert 5: And my information sources also included, as someone already mentioned, the data from the [State] Environmental Service on water, soil, and food.
Main challenges			
Time constrains	7	Eksperts 7: Tas, ka man pietrūka laika iedziļināties, izprast...	Expert 7: What I lacked was time to delve deeper and fully understand...
		Eksperts 3: Tomēr laikam, ja es skatos par laika taupīšanām, man šķistu, ka tas [eksperts], kas vāc informāciju, arī rēķina Hanlonu [Hanlona punktu skaitu].	Expert 3: However, if I think about saving time, it seems to me that the expert collecting the information should also calculate the Hanlon score.
		Eksperts 1: Mums bija tā testēšanas fāze, bija iespēja kaut ko pamainīt, kaut ko pierediģēt. Bet vienkārši viņa pagāja, jo ne visi viņu izmantoja.	Expert 1: We had the testing phase, with the opportunity to adjust or edit something. But it just passed by because not everyone made use of it.
		Eksperts 2: Jā, tieši sākt runāt par atvaļinājuma laikiem un laika trūkumu, jo man tas ļoti traucēja un tas ir sāpīgs jautājums joprojām. Jo mums bija daudz, daudz citi darbi un termiņi,	Expert 2: Yes, starting to talk about vacation times and lack of time, because that really hindered me, and it is still a sensitive issue. We had so many other tasks and deadlines.
		Eksperts 5: Mana problēma bija-vienā grupā N-desmit vielas, vairāk... un nenormāli pārtraukumi starp vielām. Ļoti liels laika patēriņš nelietderīgi. Bet tas laikam ir tas, ko darīt savādāk.	Expert 5: My problem was that in one group there were dozens of substances, or even more... and huge interruptions between substances. It was an enormous waste of time. But I guess that is something to do differently next time.
Low availability of national data	5	Eksperts 6: Tur bija kaut kas laikam par svinu, iespējams, kas bija kaut kur, laikam, medījumu gaļā, vienkārši tad, ja man ir uzrakstīts 5 medījuma produktos tika novērtos svina palielināta koncentrācija, būtībā tas neko nepasaka, jo iespējams viņi ir notestējuši 20 [paraugus], bet reāli Latvijā viņi ir 2000 [nomedīti dzīvnieki], man nav to rezultātu pret ko salīdzināt.	Expert 6: There was something about lead, possibly in the meat of hunted animals. For example, if it is written that increased lead concentrations were found in 5 such products, that essentially does not say much, because maybe they tested 20 [samples], but in reality, there are 2000 [hunted animals] in Latvia. I do not have the results to compare against.
		Eksperts 3: Datu maz, kopa maza, bet no ierēdņu puses tiek pasniegts kā … problēma, bet patiesībā problēmas vispār nav.	Expert 3: There is limited data; the sample size used in the implemented studies is small... but from the officials’ side, it is presented as a problem, whereas, in reality, there is no problem at all.
		Eksperts 6: Jo mēs pilnīgi precīzi nevaram pielīdzināt [situāciju mūsu valstī] citām Eiropas valstīm, kurām ir citāds ģeogrāfiskais stāvoklis vai kaut kādi procesi, kas notiek.	Expert 6: Because we cannot fully equate [the situation in our country] with other European countries that have different geographical conditions or certain processes taking place.
		Eksperts 6: ... Mums nebija dati par Latviju. Ekspertam 1 jeb pesticīdiem bija dati par Latviju, bet tām [ķīmisko vielu] grupām, kas bija mūsu pārziņā, tām nebija.	Expert 6: ... We did not have data for Latvia. Expert 1, regarding pesticides, had data for Latvia, but for the [chemical substance] groups under our responsibility, there were none.
Insufficiently clear opinion of state authorities	4	Eksperts 6: Izlasot vēstules, kas ir ieteikts, man trūka pamatotas informācijas no šīm vēstulēm. … tāda nepilnīga un nepilnvērtīga informācija, kā tāds informācijas avots vairāk. Jā, kaut kur eksistē, bet nevar pilnvērtīgi piemērot punktiem, nevaru pacelt augstāk, tāpēc, ka ir konstatētas dažās grupās. Tas par tām vēstulēm, gribējās viņas pilnvērtīgākas. Man vismaz... Lai man izlasot ir skaidrs, ka tur ir uz faktiem balstīti konkrēti skaitļi, lai var saprast, cik mūsu sabiedrība konkrēti patērē vai vēl kaut ko. Ja man ir +/−, tad tas ir informatīvs.	Expert 6: After reading the letters and recommendations, I felt they lacked substantiated information. … The information was incomplete and insufficient, more like a source of general information. Yes, it exists somewhere, but it cannot be fully applied to the points or elevated further because it was identified only in certain groups. Regarding those letters, I wish they were more comprehensive. At least for me… I want to clearly understand from reading them that they are based on factual, specific figures, allowing us to comprehend how much our society consumes or other relevant details. If it is just approximate, then it is only informative.
		Eksperts 5: Tās vēstules … man neko nedeva, jo … pateikt, ka vajag noteikt [vispārīgi] ftalātus, kuros ir kaut kādi 120 vai cik ftalāti, [un nepateikt kurus].	Expert 5: Those letters … did not provide anything useful to me, because … saying that phthalates need to be identified [in general], when there are about 120 or so phthalates, [without specifying which ones].
Complex and huge work	4	Eksperts 5: Kaut gan mums tur metodē bija atstrādāts, cik procenti populācijas tur... bet ne vienmēr varēja tā baigi skaidras atbildes dabūt...	Expert 5: Although we had it worked out in the methodology, the percentage of the population, etc., it was not always possible to get very clear answers...
		Eksperts 1: Es zinu, ka mēs sākotnēji gribējām vienkāršoti, bet tad, kad sākām vērtēt, tad sapratām, ka ar to vienkāršo nevaram novērtēt, kur nu vēl pēc tam kādam citam paskaidrot, ka ir tā un nevis savādāk.	Expert 1: I know that initially, we wanted to keep it simple, but once we started evaluating, we realized that the simple approach was not enough to assess the situation—let alone explain to someone else why it is the way it is and not otherwise.
		Eksperts 2: Bet te gribu piebilst, ka mums ar Ekspertu 5, nezin vai būtu vieglāk, jo mums nebija viena viela, bet mums bija vielu grupas, un mēs jau ar Ekspertu 5 pašas sev taisījām tādus seminārus, sarunas, un tad mēs pašas kaut kā mēģinājām saprast, kā mums iet un kā rakstīt tālāk.	Expert 2: But I want to add here that for Expert 5 and me, I am not sure it would have been any easier, because we did not have just one substance; we had substance groups. And Expert 5 and I organized our own seminars and discussions, trying to figure out how we were progressing and how to proceed with the writing.
		Eksperts 5: Tas [Hanlona metodoloģijas] testēšanas periods ... es pateikšu godīgi, mums līdz pēdējai grupai bija testēšanas periods, jo grupa no grupas atšķiras, tur ir visādas nianses, tur ir apakšgrupas iekš grupas, tā kā testēšana grupām ir diezgan sarežģīta. Un man nav šobrīd atbildes, kā to labāk darīt.	Expert 5: The testing period [of Hanlon methodology] … I will be honest, for us, the testing period lasted until the very last group, because groups differ from one another. There are all sorts of nuances, and there are subgroups within groups, so testing for the groups is quite complex. And at the moment, I do not have an answer on how to do it better.
		Eksperts 3: … To mežonīgo darbu, adaptējot …. novērtēšanas procesu.	Expert 3: … That overwhelming work of adapting the … evaluation process.
		Eksperts 1: Tas bija ļoti monotoni, kaut kādā brīdī apnicīgi, ... Ne dēļ informācijas trūkuma, kā citiem kolēģiem, bet man vienkārši apnika to darīt....	Expert 1: It was very monotonous, at some point even tiresome... Not due to a lack of information, like some of my colleagues experienced, but I simply got tired of doing it...
Quality control	4	Eksperts 8: Ja informāciju vāc viens un Hanlonu rēķina cits, tad no manas puses... man bija sajūta, ka ir dubulta kontrole, ka Eksperts 6 pirms manis, piemēram, izķer... Nu es nevaru sarēķināt, jo man trūkst kaut kas ... Man no tā likās, ka tas fails, tas aprēķins kļuva kvalitatīvāks un saturīgāks un loģiskāks. Tomēr šajā procesā, ja Tu šai grupai, piemēram, metāliem rēķini viens, tad tur parādās sistemātiskās kļūdas, ko mēs paldies dievam arī uzķērām. Tas ir – ja Tu kaut kādā vienā veidā kļūdies aprēķinos, tad Tu kļūdies tā uz visām grupas vielām.	Expert 8: If one person collects the information and another calculates the Hanlon score, from my perspective... I felt like there was double-checking, as Expert 6, for example, would catch something before me... I could not calculate because I was missing something... To me, it seemed that the file, the calculation, became more qualitative, substantial, and logical. However, in this process, if, for example, the calculations for a group like metals are done by one person, systematic errors can appear, which, thankfully, we also caught. That is, if you make a mistake in one specific way during the calculations, you end up making that same mistake for all the substances in the group.
		Eksperts 8: Savukārt, ja raksta ... visu dara viens, tad no kontroles.... no gala produkta kontroles viedokļa ir daudz grūtāk, jo tad tās kļūdas aprēķins, tās, ko es ar savām prasmēm un zināšanām un sistemātiku varēju pamanīt, viņas varēja būt absolūti dažādas, uz visām pusēm. Tu to pārrēķini, bet tas liecina, ka ir jābūt vairākpakāpju aprēķinu kontrolei.	Expert 8: On the other hand, if one person writes and does everything themselves, then from a control perspective... from the perspective of final product control, it is much harder. Errors in calculations, the ones I could notice with my skills, knowledge, and systematic approach, could be absolutely varied and scattered in all directions. You recalculate it, but it shows that there needs to be multi-level control of the calculations.
		Eksperts 5: Man trūka, ka mums nebija vēl kāds ķīmiķis, kas grupā pārbaudītu, vai [novērtējums] ir ok.	Expert 5: I felt the lack of having another chemist in the group to check whether it [the evaluation] was okay.
Changes in the methodology	3	Eksperts 7: Man bija prieks, ka to metodoloģiju mainīja un precizēja visu laiku, un pielāgoja, par ko es saprotu, ka raksti tiks rakstīti. ... Tā kā es esmu priecīga, ka metode ir paplašināta.	Expert 7: I was glad that the methodology was constantly updated, refined, and adjusted, which I understand will be reflected in research articles. So, I am happy that the method has been expanded.
		Eksperts 8: Viņa [metode] diezgan daudz mainījās tajā procesā.	Expert 8: It [the method] changed quite a lot during the process.
		Eksperts 2: Mēs strādājām-prioritizējām vielas tajā pašā laikā, kad Eksperts1 metodoloģiju izstrādāja, jā, tāpēc bija grūti.	Expert 2: We were working—prioritizing substances—at the same time that Expert 1 was developing the methodology, so it was challenging.
Insufficient specific knowledge	4	Eksperts 3: … jo katram ir savas bāzes zināšanas, tas, ka varbūt ir jābūt kaut kādām vadlīnijām, kādām zināšanām ir jābūt, lai tu veiktu šo nu... [ novērtējumu].	Expert 3: ... because everyone has their own base knowledge, perhaps there should be some guidelines on what knowledge is necessary to perform this... [evaluation].
		Eksperts 3: Mums ir trakoti maz to …. to toksikologu, to vides toksikologu, kas spētu šito sašķirot...	Expert 3: We have an incredibly small number of ... toxicologists, environmental toxicologists, who could sort this out...
		Eksperts 1: Vai kāds speciālists komandā trūka? … būtu forši, ka būtu kāds gudrāks kā es, kas to, ko es uzrakstu, tā saturiski forši iziet cauri. Ķīmija, toksikoloģija, mums bija pieaicināts eksperts, nu viņš kaut ko iekomentēja–Eksperts 10, bet ... jā, ja būtu vēl kāds, kas ar tādu labu bagāžu vēl būtu pārskatījis, būtu vēl labāk.	Expert 1: Was there a specialist missing from the team? … It would have been great to have someone smarter than me who could thoroughly review what I wrote in terms of content. In chemistry and toxicology, we had an invited expert—Expert 9—who made some comments, but ... yes, if there had been someone else with solid expertise to review it further, it would have been even better.
Objectivity	4	Eksperts 3: Ja to visu [Hanlona punktus] rēķina viens [ eksperts], tā pragmātiski, ņemot tos punktus, kas kurā vietā ielikti, ar vienu pieeju, vienu noteiktu pieeju visai grupai, tad ir viena noteikta standartnovirze. Ja dara dažādi cilvēki, tad tur ir subjektīvais....	Expert 3: If all [Hanlon scores] are calculated by one [expert], pragmatically, using the same approach and methodology for the entire group, then there is a consistent standard deviation. If different people do it, then there is a subjective element...
Positive aspects			
Team approach	8	Eksperts 8: Un es priecājos, ka tajā ziņā mēs bijām kā komanda, kurā viens vairāk iedziļinājās izstrādē, cits aprakstīšanā, cits vēl kaut ko darīja.	Expert 8: And I am glad that, in that sense, we worked as a team, where one person focused more on development, another on writing, and someone else handled other tasks.
		Eksperts 2: Mums ar Ekspertu 5 bija sadalīts darbs... Es liku iekšā visas vielas pēc saraksta, CAS numurus, bīstamības un tad es pildīju iekšā Hanlon B un C tabulu.	Expert 2: Expert 5 and I had divided the work... I entered all the substances from the list, CAS numbers, and hazards, and then I filled in the Hanlon B and C tables.
		Eksperts 2: Pāris reizes izlasu metodiku, un pēc tam mēģināju … izrunāt ar kolēģiem, kas nodarbojas ar to pašu.	Expert 2: I read the methodology a couple of times and then tried to discuss it with colleagues who were working on the same thing.
		Eksperts 2: Komandas gars ir ļoti labs.	Expert 2: The team spirit is very good.
		Eksperts 3: Nu mums bija tā pieeja, ka datus vāca viens, bet Hanlonu rēķināja cits.	Expert 3: Well, our approach was that one person collected the data, but another calculated the Hanlon score.
		Eksperts 7: Jā, vai informāciju var vākt un Hanlonu rēķināt cits? Noteikti! Tāpēc, ka es esmu viens no tiem cilvēkiem, kurš piedalījās ar ne pārāk lielu eksperta bagāžu, un es šajā projektā vairāk esmu tas klikšķinātājs. Un es biju priecīga, ka varu saklikšķināt un biju priecīga, ka manā darbā Hanlonu aprēķināja cits un šis cits … saprata, kur man kaut kas pietrūkst, lai es varu vēl ieklikšķināt un pamainīt.	Expert 7: Yes, can one person collect the information and another calculate the Hanlon score? Absolutely! Because I am one of those people who participated without a very extensive expert background, and in this project, I was more of a “clicker.” I was happy that I could do the clicking and glad that someone else calculated the Hanlon score based on my work. This person understood where I was missing something, so I could go back, add, and make adjustments.
		Eksperts 6: Es arī nebiju no 0 [paša sākuma] …. Eksperts 1 bija blakus un pastāstīja, kā tas notiek. Ja nu kas, es varēju pajautāt.	Expert 6: I also wasn’t starting from scratch... Expert 1 was there to explain how it works. If anything came up, I could ask.
		Eksperts 3: Kas procesā patika? ... Tas ka komanda var saliedēties, tīri raugoties kā tas ir sanācis, ka ir mežonīgi daudz panākts un izdarīts.	Expert 3: What did I like about the process? ... That the team could become closer, and looking at it now, it’s impressive how much was achieved and accomplished.
		Eksperts 4: Speciālisti bija, paldies kolēģiem, kas vienmēr bija un laipni palīdzēja. Vienmēr atbildēja, gan pētnieki, gan ķīmiķi, vienmēr visi atbildēja. Ja bija kādi jautājumi. Tas arī patika procesā. Kolēģu atsaucība.	Expert 4: The specialists were there—thanks to the colleagues who were always available and willing to help. They always responded, whether it was researchers, or chemists; everyone always answered if there were any questions. That’s something I also liked about the process—the responsiveness of colleagues.
		Eksperts 7: Būt kā tas klikšķinātājs vai tas gudri apmācītais mērkaķis, to es darītu labprāt. Jo man patīk tā sajūta ... Nu ka tu vari izdarīt kā zobratiņš tajā lielajā sistēmā priekš lieliem ekspertiem vai priekš zinošiem ekspertiem...	Expert 7: Being the “clicker” or the cleverly trained monkey—that I’d gladly do. I enjoy the feeling... that you can contribute as a small cog in the big system for the great experts or knowledgeable specialists...
Availability of the written methodology and supporting templates	6	Eksperts 6: Skatoties tos avotus, kas bija minēti [dokumentu sagatavēs].	Expert 6: Looking at the sources that were mentioned [in the document templates].
		Eksperts 7: Un Vācijas biomonitoringa normas, kas jau bija iedotas–tas bija tas labais, ka nebija jāmeklē. Varēja arī apskatīties. Man kā nespeciālistam tas ļoti palīdzēja.	Expert 7: And the German biomonitoring standards that were already provided—this was the good part, that there was no need to search for them. They could also be reviewed. For me, as a non-specialist, this was very helpful.
		Eksperts 1: Jo es ļoti gāju pēc templatiem, no sākuma bija tā aprakstošā daļa, tad es gāju B, C atsevišķi, arī tur es izmantoju tos templates, kas bija sagatavoti. Man diezgan raiti gāja uz priekšu, jo es gāju, cik punkti kam piederēja.	Expert 1: I really followed the templates. First, there was the descriptive part, then I moved on to B and C separately, and I also used the templates that were prepared. I made good progress because I followed which points belonged to which.
		Eksperts 5: Tika izmantotas arī tās listes, kas mums metodikā ir atstrādātas, mums pat bija saites, par to paldies Ekspertam 1. Tā tabula arī bija maksimāli vienkāršoti uztaisīts, lai var ieiet un to vielu meklēt uzreiz.	Expert 5: We also used the lists that were developed in our methodology, and we even had links for that—thanks to Expert 1. The table was made as simple as possible, so you could go in and search for the substance right away.
		Eksperts 7: Un es arī nezināju uzreiz, kur to informāciju meklēt.... Tādēļ man palīdzēja tie templeiti, kas visur tur bija, tas man ļoti palīdzēja...	Expert 7: And I didn’t immediately know where to search for that information... That’s why the templates that were available everywhere really helped me.
		Eksperts 2: Pāris reizes izlasu metodiku, un pēc tam mēģināju visu saprast …	Expert 2: I read the methodology a couple of times, and then I tried to understand everything...
Obtained new skills and knowledge	3	Eksperts 4: …par prioritizēšanu, es šajā reizē mācījos. Gāju visam cauri mācoties.	Expert 4: ... Regarding prioritization, I learned this time. I went through everything while learning.
		Eksperts 6: Man arī patika palasīt visādus pētījumus, kurus izlasot, es vispār saprotu, ka šeit man tas nenoder, bet bija interesanti. Un bija lietas, ko Tu uzzini jaunu.	Expert 6: I also enjoyed reading various studies, and after reading them, I realized that they didn’t really help me here, but it was interesting. And there were things that I learned anew.
Use of artificial intelligence	2	Eksperts 6: Es diemžēl arī neizmantoju, jo es tajā brīdī par to neiedomājos. Jo, ja man šobrīd jautātu, tad es noteikti izmantotu. Jo pēc tam, kad es taisīju PARCam kaut kādām vielām izvērtējumu, tur par kaut kādiem normatīviem un vēl visu kaut ko, es sapratu, ka ļoti labs rīks, kas tev iedod kaut kādu skatu punktu, kur var pamēģināt, bet jā, diemžēl neizmantoju.	Expert 6: Unfortunately, I didn’t use it either, because I didn’t think about it at the time. But if I were asked now, I would definitely use it. Because later, when I was doing the PARC evaluation for certain substances, regarding regulations and other things, I realized it is a very good tool that gives you a perspective to try out. But yes, unfortunately, I did not use it.
		Eksperts 1: Jā, es esmu... Es kaut kādā pusceļā man liekas sāku izmantot, maksas versiju. Ko es jautāju-jautāju jebko, ko es nesapratu... Vai es viņam, piemēram, prasīju, kādai ... vai tādai un tādai vielai pastāv.... kādas pastāv īpašības, un ja jā, tad ... Nu visam ko tu raksti, iedod man references. Reizēm viņš iedeva galīgi nepareizus linkus, reizēm viņš iedeva uz labiem rakstiem saites. Nu tur nav vienos vārtos un viennozīmīgi, bet bija vietas, kur viņš viennozīmīgi varēja man palīdzēt. Es atradu avotus, kādus es iepriekš nebūtu atradusi.	Expert 1: Yes, I... I think I started using the paid version halfway through. What I asked—I asked anything I didn’t understand... For example, I asked him about the properties of a certain substance, and if they exist, then... Well, for everything you write, give me references. Sometimes he gave completely incorrect links, and sometimes he gave links to good articles. It was not always clear-cut, but there were places where he could definitely help me. I found sources that I would not have found otherwise.
Suggestions for improvements			
Kick-off training and regular updates	5	Eksperts 7: Sākumā būtu labi, ka ir tās ekspertu grupas [par specifiskām vielām]. … ka visi var izrunāt, … kā grupas strādās, ... kur meklē. Tad droši vien arī būtu vienkāršāk to darīt.	Expert 7: At the beginning, it would be good to have expert groups [focused on specific substances] ... so everyone can discuss ... how the groups will work ... and where to look for information. That would probably make the process easier.
		Eksperts 6: Ir forši, ja sākotnēji visiem ir prezentācija, tad mēs izejam cauri katrs pa savam, izrunājam, lai mums ir vienota pieeja visām lietām.	Expert 6: It is great if there is a presentation for everyone at the beginning, then we each go through it individually and discuss it to ensure we have a unified approach to everything.
		Eksperts 2: Mēs jau ar Ekspertu 5 pašas sev taisījām tādus seminārus, sarunas, un tad mēs pašas kaut kā mēģinājām saprast, kā mums iet un kā rakstīt tālāk.	Expert 2: Expert 5 and I organized our own seminars and discussions, trying to figure out how we were progressing and how to proceed with the writing.
		Eksperts 1: Mēs varējām iefilmēt kādu videomateriālu, kā to dara. Pat ja mums ir tāda situācija, ka kaut kādā punktā X pamainās kolēģi, tad vienkārši Tev ir darba pakete–var iepazīties te, var noskatīties te.... Varbūt tas … kaut kā palīdzētu.	Expert 1: We could have recorded a video tutorial on how it is done. Even if we find ourselves in a situation where colleagues change at some point, you would simply have a work package—everything is explained here, and you can watch it here. Maybe that... could help in some way.
		Eksperts 2: Principā esmu priecīga, ka mēs ar Ekspertu 5 strādājām komandā, un mēs sarunājāmies un mums bija tādi nelieli brainstormingi, dažreiz tie ilga kādas stundas 2, tas man ļoti palīdzēja. Varbūt šoreiz es, ja es vēlreiz tādam piekristu, es gribētu tādus brainstormingus darīt biežāk, lai izrunātu, lai visas savas emocijas pateiktu, un... dalītos pieredzē ar kolēģiem.	Expert 2: Overall, I am happy that Expert 5 and I worked as a team. We had discussions and small brainstorming sessions, sometimes lasting about two hours, which helped me a lot. If I were to agree to do something like this again, I would want to have such brainstorming sessions more frequently—to discuss, share all my thoughts and emotions, and exchange experiences with colleagues.
Previous involvement in international projects	3	Eksperts 1: Ir grūti to programmu no zila gaisa izveidot, tāpēc mans padoms būtu-rast veidu, kā iesaistīties kaut kādās sistēmās, lielajos Eiropas projektos. Jo tas, ka ir bijis HBM4EU, tas ka ir PARC, tas tomēr palīdz to.... Arī to ideju saprast, nevis, ka Tu lasi publikāciju un mēģini uzburt, tomēr tā citu valstu tiešā pieredze, ko Tu vari iegūt no partneriem ir tāda riktīgi vērtīga.	Expert 1: It is difficult to create a program out of thin air, so my advice would be to find a way to get involved in some systems or major European projects. The existence of HBM4EU and PARC really helps with that... It also helps to understand the concept, rather than just reading publications and trying to imagine it. The direct experience of other countries, which you can gain from partners, is truly valuable.
		Eksperts 6: Es piekrītu laikam Ekspertam 1, ka jāiesaistās citās ārpus programmās vai aktivitātēs, vai iespējams, jāatrod kolēģi no citām valstīm, kas arī to ir sākuši darīt. Sākumā ņemt kādu piemēru, lai tev ir kāds pamata materiāls, uz ko balstīties, un tad visticamāk ir vieglāk.	Expert 6: I probably agree with Expert 1 that it’s necessary to get involved in other external programs or activities, or perhaps find colleagues from other countries who have also started working on this. Initially, take an example to have some foundational material to build upon, which would likely make things easier.
		Eksperts 2: Kāds varētu būt padoms? … Laikam kaut kā palasīt kā citi ir veikuši tādu projektu. Kādas bija problēmas? Varbūt, ka būs labi, ka būtu tāds raksts pārdomām-kādas bija priekšrocības, kādi bija trūkumi.	Expert 2: What advice could there be? ... Perhaps to read about how others have carried out similar projects. What problems did they encounter? Maybe it would be useful to have an article reflecting on the advantages and shortcomings.
Change management	2	Eksperts 8: Es gribētu, lai procesa gaitā nemainītos komanda, kas nav ietekmējams, jo komanda var mainīties ļoti dažādu apstākļu dēļ. Jo arī liela daļa no tā, ko jūs minējāt, bija tas, ka “Es ielecu vēlāk tajā procesā”.	Expert 8: I would prefer that the team doesn’t change during the process, although this isn’t always controllable, as the team can change due to various circumstances. A large part of what you mentioned was also that “I joined the process later”.
		Moderators: … diez vai ir kāda valsts, kāda situācija, kāds projekts, kas notiek pilnīgi nenormāli gludi. Tāpēc ir pilnīgi normāli, ka kāds ielec, kāds izlec...	Moderator: ... It is unlikely that there’s any country, situation, or project that runs completely seamlessly. So, it is entirely normal for someone to jump in and someone else to step out...
		Eksperts 8: Mans lielākais izaicinājums visā šajā procesā par metodoloģijas izstrādi bija tiešām, panākt to, ka situācijā, kad metodoloģija drusciņ pamainās, ka eksperti tās ievēro.	Expert 8: My biggest challenge in this entire process of developing the methodology was ensuring that, in situations where the methodology slightly changes, that experts fulfil them.
Clear state framework	2	Eksperts 8: Es laikam teiktu, ka valsts biomonitoringa programmas izveide ir tīri praktisks process un tas ir jāatdala no zinātnes. No valsts institūcijām un valstij kā tādai ir vienas vajadzības, un mēs jau arī šodien dzirdējām, ka zinātniekiem un pētniekiem ir pilnīgi citas vajadzības un intereses.	Expert 8: I would say that establishing a national biomonitoring program is a purely practical process and should be separated from science. State institutions and the state itself have one set of needs, while, as we heard today, scientists and researchers have completely different needs and interests.
		Eksperts 7: Man … būtu gribējies, ka tās valsts iestādes būtu vairāk iedziļinājušās, ieinteresētas, ka mums jau vairāk iedotu. Nevis kaut kā pētniekiem interesanti pētīt, bet iedod rāmi–“O! Nu to vajadzētu papētīt.”	Expert 7: I would have liked for the state institutions to have been more engaged and interested, providing us with more input. Not just something researchers find interesting to study, but giving a framework—”Oh! This is something that should be investigated.”
